# Post-Pancreatectomy Acute Pancreatitis in Pancreatic Cancer: A Single-Center Experience

**DOI:** 10.3390/medicina62091788

**Published:** 2026-09-16

**Authors:** Ewa Grudzińska, Magdalena Gajda, Karolina Majewska, Wojciech Dubaj, Sławomir Mrowiec

**Affiliations:** Department of Gastrointestinal Surgery, Medical University of Silesia, 40-055 Katowice, Poland; magdalena.gajda@sum.edu.pl (M.G.); majewskakarolina1008@gmail.com (K.M.); w.dubaj90@gmail.com (W.D.); smrowiec@sum.edu.pl (S.M.)

**Keywords:** acute pancreatitis, pancreatectomy, pancreatic surgery, post-pancreatectomy acute pancreatitis

## Abstract

*Background and Objectives*: In 2022, the International Study Group of Pancreatic Surgery (ISGPS) introduced a definition of postoperative acute pancreatitis (PPAP). This study aimed to assess the perioperative factors associated with clinically relevant PPAP (CR-PPAP) after pancreatoduodenectomy (PD) due to pancreatic ductal adenocarcinoma (PDAC) and to present the real-world treatment strategies for this complication. *Materials and Methods*: We performed a retrospective single-center analysis of 282 patients who underwent pancreatoduodenectomy for PDAC between 2010 and 2023. *Results*: CR-PPAP was diagnosed in 13.1% of patients. This group had significantly higher CRP (219 vs. 42 mg/L) and more frequently elevated drain amylase activity (97.3% vs. 18%). CR-PPAP was associated with significantly higher rates of reoperation (37.8% vs. 3.3%), delayed gastric emptying (73% vs. 16.3%), hemorrhage (21.6% vs. 0.8%) and pancreatic fistula (97.3% vs. 0.8%), as well as longer hospitalization (15 vs. 13 days). Antibiotics were administered significantly more often in the CR-PPAP group (86.5% vs. 6.5%), while somatostatin was used in all CR-PPAP patients and octreotide in 62.2%. Thirty-day mortality did not differ significantly between groups. In multivariate analysis, a small main pancreatic duct was the only independent factor associated with CR-PPAP. *Conclusions*: PPAP, as defined by the ISGPS 2022 criteria, is a relatively common complication following PD for malignancy, with a small pancreatic duct being a major risk factor. PPAP is strongly associated with other major postoperative complications and prolonged hospitalization, but not with increased mortality. Despite the frequent use of antibiotics, octreotide, and somatostatin in clinical practice, evidence-based treatment recommendations for CR-PPAP remain lacking, highlighting the need for management guidelines.

## 1. Introduction

Postoperative acute pancreatitis is a severe complication that can occur after surgery in the pancreatic region, such as gastric surgery or splenectomy. It also occurs in about 7.5–18.2% of patients after pancreatic surgery [1,2,3]. In 2022, the International Study Group of Pancreatic Surgery (ISGPS) classified post-pancreatectomy acute pancreatitis (PPAP) as a separate disease, defining it as inflammation of the pancreatic stump occurring in the first 3 days after partial resection of the pancreas. The diagnostic criteria are persistent elevation of serum amylase for at least 48 h after operation, correlated with the patient’s clinical deterioration and radiological changes.

ISGPS divided PPAP into three grades according to severity. Grade A is associated with biochemical changes only and has no clinical relevance. Grade B includes patients with mild or moderate complications. Grade C refers to patients with severe and life-threatening complications [4]. This single, standardized definition of PPAP allows a comparison of postoperative outcomes among medical centers, which can subsequently help to improve treatment results. The studies show that while patients with PPAP -A or -B have similar treatment outcomes as patients without these complications, the PPAP -C significantly worsens the outcomes [5]. PPAP -B and -C, which require medical intervention, are considered the clinically relevant PPAP (CR-PPAP). To date, there is no universally recommended treatment for CR-PPAP; therefore, the usual acute pancreatitis management is most often implemented. However, the postoperative character of the disease may require a different approach.

This study presents a retrospective, single-center analysis of the prevalence, treatment, and perioperative factors connected to the CR-PPAP as defined by the ISGPS, after pancreatoduodenectomy performed due to cancer. We identify factors correlated with the occurrence of CR-PPAP and present the real-life clinical approach to CR-PPAP treatment.

## 2. Materials and Methods

We retrospectively analyzed 296 patients who underwent pancreatoduodenectomy with curative intent for pancreatic ductal adenocarcinoma (PDAC) at our department between 2010 and 2023. The data were extracted from a prospectively maintained institutional database. Following data screening, 14 patients were excluded due to incomplete clinical data, and the final study cohort was 282 patients with complete data available for analysis.

**Figure 1:**
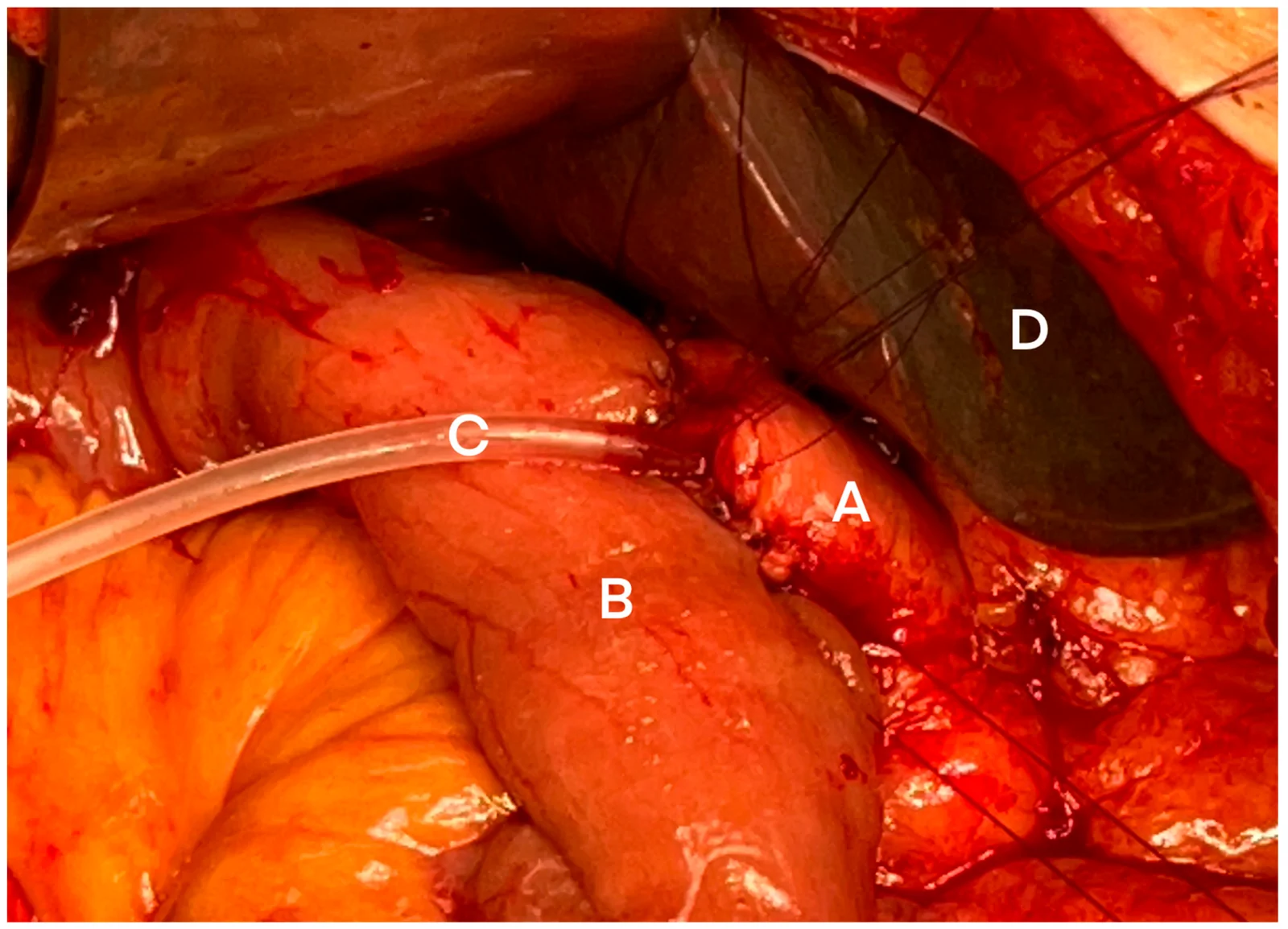
The mucosa-duct pancreaticojejunostomy in progress. A—pancreatic stump, B—jejunum, C—a drain inserted temporarily into the MPD, D—left hepatic lobe. The posterior exterior and interior parts of the anastomosis are finished, and the untied sutures are placed in the anterior wall of the MPD—the interior anterior part of the anastomosis is in progress.

All patients were operated on by a single experienced surgeon. A standard open pylorus-preserving pancreatoduodenectomy (Traverso procedure) or Whipple procedure was performed. The choice of procedure was left to the discretion of the operating surgeon and depended on pyloric involvement by the tumor. In all cases, a duct-to-mucosa pancreaticojejunostomy was performed (Figure 1). Two intra-abdominal drains were routinely placed: one near the pancreatic anastomosis and the other adjacent to the biliary anastomosis. The following variables were analyzed: demographic data (sex, age, body mass index [BMI]); preoperative parameters (American Society of Anesthesiologists [ASA] physical status classification and neoadjuvant therapy); intraoperative data (resectability status, type and duration of surgery, intraoperative blood loss, main pancreatic duct [MPD] diameter, and pancreatic texture classified as soft or non-soft); and postoperative parameters (serum amylase, lipase, and C-reactive protein [CRP] levels 48 h after surgery, drain fluid amylase activity, abnormalities detected on postoperative computed tomography [CT], reoperations, histopathological findings, length of postoperative hospital stay, postoperative antibiotic and somatostatin therapy, and 30-day mortality).

MPD diameter and pancreatic texture were assessed intraoperatively by the operating surgeon. Resectability assessment was based on the National Comprehensive Cancer Network criteria [6]. Postoperative CT was performed in patients with clinical deterioration and/or markedly elevated inflammatory markers (CRP, leukocytosis, or elevated serum amylase and lipase levels) and/or in those who developed Clavien–Dindo grade III or IV postoperative complications.

The association between the collected variables and the occurrence of CR-PPAP was assessed.

The study was conducted in accordance with the Declaration of Helsinki. As a retrospective analysis based solely on the existing, fully anonymized data, with no patient involvement, in accordance with the national legislation, the study is not considered a medical experiment. Therefore, the consent of the bioethics committee and the patient’s consent were not required.

### Statistical Analysis

Statistical analysis was performed using IBM SPSS software version 30.0. Continuous variables were reported as median and interquartile range. Categorical variables were reported as frequencies. Between-group differences were assessed with the Mann–Whitney U test for continuous variables and with the χ2 test or Fisher’s exact test for categorical variables. Multivariate logistic regression was used to assess risk factors for CR-PPAP. Potential risk factors were selected based on previous publications.

## 3. Results

The analysis included 282 consecutive patients with pancreatic cancer who underwent pancreaticoduodenectomy in our department. The demographic data and the data on perioperative factors are presented in Table 1. A total of 59.2% of patients were female. The median age was 67 (30–82), and the median body mass index (BMI) was 24 (18–41); 78.4% of patients were American Society of Anesthesiologists (ASA) class II. In total, 54.3% of the tumors had borderline resectability, and 48.2% of patients received neoadjuvant therapy. The median surgery duration was 445 min (295–615), and the median blood loss was 300 mL (200–1200). The median MPD diameter was 2 mm (0–10). Postoperatively, the serum amylase and lipase were assessed after 48 h, and the median activity was 18 U/L (0–1230) and 19 U/L (3–1450), respectively. The upper normal for amylase in our institution is 100 U/L for serum amylase and 60 U/L for serum lipase. Postoperative hyperamylasemia (POH) was found in one-third of patients; however, the CR-PPAP was diagnosed in 13.1% of patients (PPAP-B in 9.6% and PPAP-C in 3.5%).

Table 2 presents the postoperative data for patients, divided into the CR-PPAP group (*n* = 37; 13.1%) and the group without CR-PPAP (*n* = 245; 86.9%). The CR-PPAP group had significantly higher CRP 48h after surgery (median: 219 mg/L (95; 34–345) vs. 42 mg/L (30; 8–218)), significantly more often had elevated drain amylase activity (97.3% vs. 18%), and required antibiotics (86.5% vs. 6.5%).

**Figure 2:**
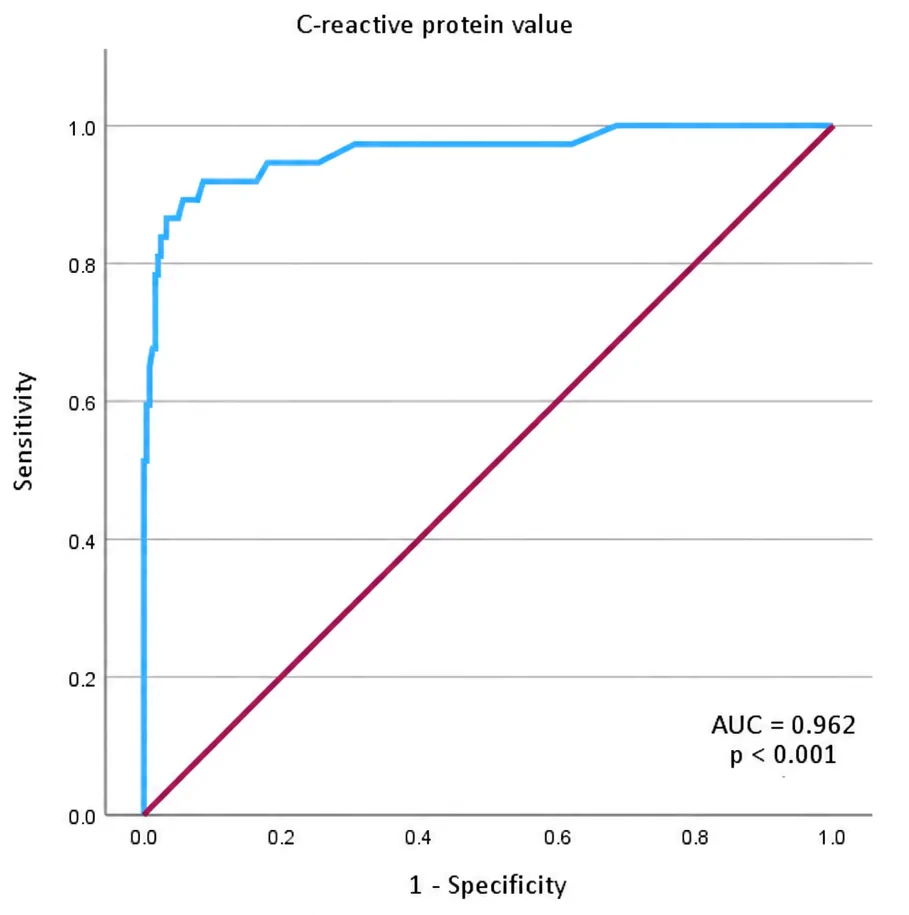
CRP level 48h after the surgery predicts the development of CR-PPAP. The optimal cut-off value is 98.5 mg/L (sensitivity 89.2%, specificity 94.3%).

The cut-off value of CRP concentration 48 h after surgery with the highest predictive value for CR-PPAP in our cohort was 98.5mg/L (sensitivity 89.2%, specificity 94.3%) (Figure 2).

All CR-PPAP patients had CT changes as required by the diagnostic criteria (intra-abdominal fluid collection, pancreatic stump edema, inflammation of the pancreatic stump, pancreatic necrosis). Some of these were also found in the no-CR-PPAP group; however, these were found significantly less often.

Somatostatin was administered only in the CR-PPAP group, in all patients.

Octreotide was administered in 62.2% of CR-PPAP patients vs. 17.1% in the no-CR-PPAP group. It did not show any significant correlation with the occurrence of severe complications, reoperations, CT changes, or hospitalization duration and 30-day mortality (Table 3).

In the CR-PPAP group, antibiotics were administered to most patients (86.5%), and these patients had significantly shorter hospital stay (14 vs. 20 days, *p* = 0.028). No significance was found considering severe postoperative complications, reoperations, CT changes or 30-day mortality (Table 4).

In the CR-PPAP group, reoperations were significantly more prevalent (37.8% vs. 3.3%); most often a completion pancreatectomy (distal pancreatectomy with splenectomy) was performed (10 of 14 reoperations in the CR-PPAP group vs none in the no-CR-PPAP group). Hospitalization was significantly longer in the CR-PPAP group (median 15 days (7; 11–38) vs. 13 days (3; 8–25)). However, reoperations did not have a significant impact on hospital stay in either group (Table 5).

The 30-day postoperative mortality did not show significant differences between the groups, with one death in the CR-PPAP group.

The neoadjuvant therapy did not show any correlation with CR-PPAP occurrence (CR-PPAP in 15.1% of patients vs 11.0% of patients in the no-neoadjuvant vs neoadjuvant groups, Table 6).

The selection of variables for multivariable logistic regression (Table 7) was based on previously reported predictors of PPAP and was limited to preoperative and intraoperative factors, including BMI, MPD diameter, sex, soft pancreatic tissue, and surgery duration. Owing to the relatively small number of CR-PPAP events, the number of covariates was intentionally restricted to reduce the risk of model overfitting.

Only the MPD diameter had a significant impact on the CR-PPAP risk. The smaller the diameter, the greater the CR-PPAP risk.

## 4. Discussion

### 4.1. PPAP Occurrence After PD

A single-center retrospective analysis by Bellotti et al. included 272 patients after PD. In 14.7% of them, PPAP occurred [2]. Ikenaga et al. analyzed a group of 247 patients after pancreaticoduodenectomy. According to the ISGPS definition, eight patients developed PPAP grade B, and one patient developed PPAP grade C [7]. Quero et al. showed that among 520 patients after pancreaticoduodenectomy, 63 developed PPAP [8]. In our cohort, PPAP was diagnosed in 13.1% of patients, which is a similar outcome. It must be noted that, as a retrospective, single high-volume center and a single surgeon’s experience, the study may present a different environment than studies conducted in low-volume centers or including surgeons with different experience levels. Also, CT was not performed routinely in all patients but according to the decision of the treating surgical team based on the patient’s postoperative clinical course. This may have caused a selection bias in the comparison of radiological findings between the CR-PPAP and no-CR-PPAP groups. However, all patients diagnosed with CR-PPAP underwent CT imaging, as radiological findings are included in the ISGPS definition of CR-PPAP, together with clinical and biochemical symptoms. In our institution, CT is routinely performed in patients with postoperative clinical deterioration, markedly elevated inflammatory markers, and/or significantly increased serum amylase and lipase levels. Therefore, it is highly unlikely that a patient meeting the criteria for CR-PPAP would not undergo CT evaluation and consequently remain undiagnosed. Nevertheless, the absence of CT examinations in some patients without CR-PPAP limits the direct comparison of postoperative radiological findings between the study groups.

### 4.2. Post-PD PPAP Risk Factors

PDAC is characterized by aggressive tumor biology, frequent lymphatic dissemination, and a high prevalence of advanced pathological features, all of which may contribute to the complexity of perioperative management and postoperative recovery [9]. Complications such as CR-PPAP should not be viewed in isolation, but rather as part of a multifactorial postoperative course in oncological pancreatic surgery, where tumor-related factors, surgical complexity, and patient condition may interact [10]. Neoadjuvant therapy, for example, has been considered in the context of postoperative complications. There is, however, no proof of worsened surgical outcomes, including the occurrence of POPF [11]. The surgical technique is also constantly evolving, including the minimally invasive approach. Laparoscopy is now considered standard for distal pancreatectomy, and robotic surgery is increasingly implemented for PD, with oncological outcomes comparable to open surgery, faster recovery rates, and, as reported by some studies, decreases in POPF rates [12,13,14].

Chen et al. determined risk factors for PPAP as female sex, robotic surgery, normal bilirubin level, nondilated main pancreatic duct, and a high-risk no-adenocarcinoma disease [15]. In a more recent publication by Chen et al., in 601 patients after PD, CRP > 100 mg/L, BMI ≥ 24, and estimated blood loss > 200 mL were found to increase CR-PPAP risk [16]. In an analysis by Quero et al., the MPD diameter of 3 mm or less and soft texture of the pancreas were identified as risk factors of PPAP [8]. In our previous analysis of 428 patients who underwent PD in our medical center within 10 years, regardless of the diagnosis, CR-PPAP was found in 18.2%. In this more varied group, the statistical analysis showed that blood loss during operation, soft pancreatic texture, small diameter of pancreatic duct, and adenocarcinoma of the papilla of Vater appeared to be significant predictors of PPAP occurrence after PD [3].

This study included only cancer patients, and in this cohort, the small pancreatic duct was the only PPAP risk factor. Neither soft pancreatic tissue nor the patient’s sex or BMI showed any correlation with PPAP occurrence. As all surgeries were performed by open surgery, in a standardized manner and by a single surgeon, the impact of the operative technique was not assessed. Preoperative bilirubin levels were not included in our analysis.

The elevated CRP levels after 48 h postoperatively are included in the ISGPS CR-PPAP definition. As concluded by Chen et al., this suggests that in post-PD patients with elevated CRP, early anti-inflammatory intervention might be beneficial. These patients may also benefit from early CT assessment and intensive monitoring.

It is worth noting that the elevated amylase activity in the drain fluid has a strong association with CR-PPAP (97.3% vs. 18%). This parameter is not included in the ISGPS criteria; however, it seems a valuable addition to the early diagnosis.

### 4.3. PPAP Prevention and Treatment

As PPAP is one of the most important post-PD complications, its prevention, early detection, and treatment remain under investigation. The therapeutic data presented in this study should be interpreted as a descriptive real-world clinical approach rather than evidence of treatment effectiveness because pharmacological treatment was administered according to clinical judgment and not by randomized allocation.

Multiple studies have analyzed somatostatin and octreotide and their role in pharmacological prevention and treatment when a high risk of PPAP is suspected or when PPAP is diagnosed. Somatostatin and octreotide administration in our cohort did not show any correlation with postoperative outcomes. It must be noted that our data do not indicate the time of drug administration. Some patients may have received pharmacological treatment before CR-PPAP occurrence due to a risky pancreaticojejunal anastomosis (soft pancreatic tissue, small MPD) and others after the onset of PPAP (biochemical or clinical symptoms). Octreotide is supposed to reduce pancreatic exocrine secretion, but the available data are not consistent. Multiple analyses did not confirm its positive impact on post-pancreatectomy complications [17,18]. Octreotide is not recommended as routine treatment or prevention considering its risks (hyperglycaemia, impaired splanchnic blood flow, impairment of immunity) [19,20]. Similarly, there are no recommendations for the use of somatostatin [21]. In search of pharmacological methods to improve PPAP treatment, Bannone E et al. studied another protease inhibitor, gabexate mesylate (GM). Unfortunately, no difference was found in PPAP occurrence in the group of patients treated with GM in comparison to the control group. Therefore, the use of GM appears to be irrelevant for PPAP prevention [22]. The combination of more than one drug—ulinastatin with somastatin—has shown some benefit in acute pancreatitis treatment, reducing acute respiratory distress syndrome, multiorgan failure, and kidney injury, while also reducing the hospital stay; however, it has not been confirmed in PPAP [23].

The universal use of somatostatin in CR-PPAP patients in our cohort reflects institutional management practice. The comparison of patients who did or did not receive octreotide is limited by non-randomized allocation, uncertain timing of administration, and likely confounding by clinical severity. Consequently, these data cannot determine whether octreotide or somatostatin modifies the course of CR-PPAP. Similar practice is described in analyses from other centers, with somatostatin, ulinastatin, and octreotide administered to most PPAP patients without providing measurable benefit [18].

In our CR-PPAP group, antibiotics were associated with a shorter median hospital stay than in untreated patients (14 vs. 20 days); however, they did not have any significant impact on severe complications, reoperation rate, or mortality of PPAP. The untreated CR-PPAP subgroup was very small (*n* = 5), which limits definitive conclusions. Moreover, because antibiotic administration was not randomized and was determined by clinical judgment, the results are vulnerable to selection bias and confounding by indication. Patients selected for antibiotic therapy may have differed from untreated patients, for example, in disease severity or suspected infection. Accordingly, our data do not prove that antibiotic therapy improves outcomes in PPAP but rather indicate that antibiotics are frequently used in real-world management, especially in patients with concomitant POPF. Prospective studies are needed to determine which CR-PPAP patients may benefit from antibiotics and under what clinical circumstances. It is worth noting that acute pancreatitis guidelines recommend antibiotics only for infected necrosis and discourage antibacterial prophylaxis [19]. PPAP, however, is very often accompanied by POPF (97.3% of CR-PPAP patients in this cohort), which may partly explain the frequent use of antibiotics in our practice. Antibiotics have been shown in previous studies to improve treatment outcomes in POPF [14].

A large meta-analysis showed that data available on multiple pharmacological interventions (ethylenediaminetetraacetic acid, gabexate, glucagon, iniprol, lexipafant, non-steroidal anti-inflammatory drugs, octreotide, oxyphenonium, probiotics, activated protein C, somatostatin, somatostatin plus omeprazole, somatostatin plus ulinastatin, thymosin, ulinastatin) are of low quality and none of these reduced short-term mortality in acute pancreatitis [24]. This shows that we still lack studies on this matter and that guidelines on PPAP treatment are needed to prevent unnecessary pharmacological interventions.

The key mechanism of PPAP is impaired blood circulation in the pancreatic remnant, causing premature activation of pancreatic enzymes and subsequently leading to necrotic changes [25]. Interestingly, in Bellotti’s study, the patients who received low-molecular-weight-heparin (LMWH) bridging therapy presented a significantly lower prevalence of PPAP. It suggests that the use of anticoagulants may play a key role in the PPAP prevention strategy [2], and prospective studies are needed to determine whether anticoagulation has a role in PPAP prevention. In our department, LMWH in prophylactic doses is administered to all patients postoperatively and, therefore, was not included in the analysis.

The inflammation in PPAP is addressed by studies on anti-inflammatory drugs, including steroids and indomethacin [26]. Their role in prophylaxis and treatment is still under investigation [27]. In a study by Radulova et al., the perioperative administration of 100 mg hydrocortisone intravenously (during surgery and for 48 h, every 8 h, in patients with high risk of POPF) did not show any impact on postoperative hyperamylasemia, PPAP, or other complications [28]. Similar results were obtained by Chen et al. [29]. In post-ERCP pancreatitis prevention, rectal administration of diclofenac or indomethacin is included in the recommendations [30]; it is not a routine prophylaxis after surgery. In our department, anti-inflammatory drugs are not administered routinely.

Appropriate nutrition is crucial for postoperative recovery and for acute pancreatitis treatment. The acute pancreatitis 2024 guidelines recommend early oral nutrition, when possible, with a solid low-fat diet [31]. In a postoperative setting, the peristaltic paralysis, as well as DGE, may additionally impair oral nutrition. At the same time, the elevated catabolism caused by operative trauma and healing increases the need for an appropriate diet. We did not analyze our patients’ nutrition; however, in any case, when oral feeding is impossible for 5 days or more, parenteral nutritional support is administered, and for patients who tolerate oral fluids, oral nutritional support is administered routinely. It is worth noting that pancreatic exocrine insufficiency is common after pancreaticoduodenectomy and in pancreatitis and can lead to malnutrition and fat-soluble vitamin deficiencies [32,33]. Some patients may therefore benefit from pancreatic enzyme supplementation, once oral nutrition is implemented [34]. The fecal elastase test is useful for the assessment of pancreatic exocrine insufficiency; however, it is not routinely performed after PD [35].

The most common reoperation in the CR-PPAP group was completion pancreatectomy, i.e., removal of the remaining pancreas with splenectomy. It is a high-risk procedure, leading to postoperative diabetes and severe metabolic changes, and it is not recommended routinely for this condition [36]. However, in some patients, early removal of the leaking pancreaticojejunal anastomosis seems to be the best option. Postoperative hyperamylasemia on POD1 was shown to have a predictive value for rescue completion pancreatectomy in an analysis of 437 patients after PD [37]. Reoperation did not significantly prolong the hospital stay in any group, suggesting that in this group of patients, contrary to the patients with other types of acute pancreatitis, it may be a crucial intervention, addressing other postoperative complications connected to PPAP, such as POPF [3,31].

### 4.4. PPAP Consequences

A single-center retrospective analysis by Bellotti et al. included 272 patients after PD. In 14.7% of them, PPAP occurred, which was related to a higher risk of POPF and post-pancreatectomy hemorrhage, and consequently led to longer hospital stay [2]. Quero et al. similarly showed that the incidence of PPAP grade B and C was correlated with POPF, hemorrhage, or DGE [8].

In our study, CR-PPAP patients also had significantly more often CR-POPF, DGE, and hemorrhage, and required a longer hospital stay. CR-POPF was present in almost all CR-PPAP patients, indicating a strong association between the two complications. The frequent complications, the more prevalent use of antibiotics, and the reoperation occurrence in these patients indicate that CR-PPAP deteriorates the postoperative course and must also have a negative impact on treatment costs, although this was not assessed in our study. In other studies, hospitalization costs of patients with PPAP were 2 times greater for PPAP grade B and 11 times greater for PPAP grade C in comparison to patients without postoperative complications [1].

In accordance with other available studies, CR-PPAP did not influence the postoperative 30-day mortality. PPAP combined with POPF, however, was shown by some to increase mortality [26].

### 4.5. Study Limitations

Our study has several limitations. It was a retrospective cohort study with potential selection bias. Fourteen patients were excluded because of incomplete clinical data. Despite the relatively large overall cohort, the CR-PPAP subgroup was small, which may cause residual confounding in the multivariable analysis. The regression model was deliberately restricted to selected preoperative and intraoperative variables, and inclusion of a larger number of covariates could have caused model overfitting and unstable estimates. Some clinically relevant variables, such as blood loss, neoadjuvant therapy, inflammatory markers, and postoperative complications, were not included in the final model or were analyzed separately. Also, the amylase serum levels were not systematically measured in all patients on the first postoperative day; therefore, the elevated concentrations at 48 h were used for the PPAP diagnosis, along with the clinical and radiological findings. In the ISGPS PPAP definition, however, a sustained elevation is required, which is best documented by measurements both on the first and second postoperative days.

Because the treatments used were not randomly assigned, conclusions regarding treatment effectiveness should be considered descriptive and hypothesis-generating rather than evidence of therapeutic benefit. Although all procedures were performed by a single surgeon, the surgical approach to PDAC evolved over the long inclusion period, which may have contributed to some variability in operative and postoperative management. In addition, the single-center, single-surgeon design limits external validity and may reduce comparability with other centers. Postoperative care, patient monitoring, and imaging methods also changed during the study period. In our cohort, PPAP and POPF overlapped substantially, which may have acted as an additional confounding factor. Finally, standards for diagnosing PPAP were not uniform before the publication of the ISGPS definition, making comparisons with earlier studies more difficult.

## 5. Conclusions

PPAP, according to the ISGPS 2022 definition, is a relatively common complication of PD due to cancer, with small pancreatic duct diameter being an important risk factor. It is associated with prolonged hospital stay, additional imaging diagnostics, and a higher rate of reoperations. Although patients treated with antibiotics had a shorter hospital stay in this cohort, this association should not be interpreted as evidence of a causal benefit, given the small untreated subgroup and potential confounding by indication. Similarly, the frequent use of somatostatin and octreotide reflects real-world clinical practice, but their efficacy cannot be established from these non-randomized data. Prospective studies and clear treatment guidelines are urgently needed, as management strategies established for other forms of acute pancreatitis may not be fully applicable in the postoperative setting.

## Figures and Tables

**Table 1:** General characteristics of the analyzed population.

Variable	Median (IQR, Range) or *n* (%)
Female sex (*n*, %)	167 (59.2%)
Age (years)	67 (11; 30–82)
BMI (kg/m^2^)	24 (4.5; 18–41)
ASA class	
I	32 (11.3%)
II	221 (78.4%)
III	29 (10.3%)
Borderline tumor	153 (54.3%)
Resectable tumor	129 (45.7%)
Neoadjuvant chemotherapy	136 (48.2%)
Type of PD	
-Whipple	157 (55.7%)
-Traverso	125 (44.3%)
Duration of the surgery (minutes)	445 (70; 295–615)
Blood loss (mL)	300 (100; 200–1200)
MPD diameter (mm)	2 (1; 0–10)
Serum amylase level 48 h after surgery (U/L)	18 (138; 0–1230)
Serum lipase level 48 h after surgery (U/L)	19 (151; 3–1450)
POH	86 (30.5%)
-POH—only biochemical changes	49 (17.4%)
CR-PPAP	37 (13.1%)
-PPAP-B	27 (9.6%)
-PPAP-C	10 (3.5%)

**Table 2:** Postoperative course in patients with and without CR-PPAP.

Variable	Total (*n* = 282)	CR-PPAP (*n* = 37, 13.1%)	No CR-PPAP (*n* = 245, 86.9%)	*p*
CRP 48 h after surgery (mg/L)	45 (41; 8–345)	219 (95; 34–345)	42 (30; 8–218)	<0.001
Elevated drain amylase	80 (28.4%)	36 (97.3%)	44 (18%)	<0.001
48 h after surgery				
CT changes after surgery				
-intra-abdominal fluid collection	54 (19.1%)	36 (97.3%)	18 (7.3%)	<0.001
-pancreatic stump edema	45 (16%)	32 (86.5%)	13 (5.3%)	<0.001
-inflammation of the pancreatic stump	29 (10.3%)	28 (75.7%)	1 (0.4%)	<0.001
-pancreatic necrosis	6 (2.1%)	5 (13.5%)	1 (0.4%)	<0.001
Delayed gastric emptying (yes)	67 (23.8%)	27 (73%)	40 (16.3%)	<0.001
CR-POPF (yes)	38 (13.5%)	36 (97.3%)	2 (0.8%)	<0.001
Hemorrhage (yes)	10 (3.5%)	8 (21.6%)	2 (0.8%)	<0.001
Antibiotics administration (yes)	48 (17%)	32 (86.5%)	16 (6.5%)	<0.001
Sandostatin administration (yes)	65 (23%)	23 (62.2%)	42 (17.1%)	<0.001
Somatostatin administration (yes)	37 (13.1%)	37 (100%)	0	<0.001
Duration of hospitalization after surgery (days)	13 (3; 8–38)	15 (7; 11–38)	13 (3; 8–25)	<0.001
Reoperation (yes) - distal pancreatectomy with splenectomy - drainage of a hematoma - suturing of biliary anastomosis - drainage of intra-abdominal fluid collections - repair of evisceration - repair of evisceration with drainage of intra-abdominal collections - reduction of the intussuscepted bowel loop - retroperitoneal space abscess drainage	22 (7.8%) 10 (3.5%) 3 (1.1%) 2 (0.7%) 2 (0.7%) 2 (0.7%) 1 (0.4%) 1 (0.4%) 1 (0.4%)	14 (37.8%) 10 (27%) 1 (2.7%) 0 2 (5.4%) 0 0 0 1 (2.7%)	8 (3.3%) 0 2 (0.8%) 2 (0.8%) 0 2 (0.8%) 1 (0.4%) 1 (0.4%) 0	<0.001
30-day mortality	1 (0.4%)	1 (2.7%)	0	0.131

**Table 3:** Postoperative course according to octreotide administration.

	Octreotide—Yes (*n* = 23, 62.2%)	Octreotide—No (*n* = 14, 37.8%)	*p*
Severe complications (CD grade 3–5)	9 (39.1%)	8 (57.1%)	0.286
Reoperation (yes)	8 (34.8%)	6 (42.9%)	0.623
CT changes after surgery			
- intra-abdominal fluid collection	22 (95.7%)	14 (100%)	1.000
- pancreatic stump edema	20 (87%)	12 (85.7%)	1.000
- inflammation of the pancreatic stump	17 (73.9%)	11 (78.6%)	1.000
- pancreatic necrosis	3 (13%)	2 (14.3%)	1.000
Duration of hospitalization after surgery (days)	15 (3, 11–23)	19 (9, 12–38)	0.257
30-day mortality	1 (4.3%)	0	1.000

**Table 4:** Postoperative course according to antibiotic administration in the CR-PPAP group.

	Antibiotics—Yes (*n* = 32, 86.5%)	Antibiotics—No (*n* = 5, 13.5%)	*p*
Severe complications (CD grade 3–5)	14 (43.8%)	3 (60%)	0.644
Reoperation (yes)	11 (34.4%)	3 (60%)	0.346
CT changes after surgery			
- intra-abdominal fluid collection	31 (96.9%)	5 (100%)	1.000
- pancreatic stump edema	27 (84.4%)	5 (100%)	1.000
- inflammation of the pancreatic stump	24 (75%)	4 (80%)	1.000
- pancreatic necrosis	3 (9.4%)	2 (40%)	0.126
Duration of hospitalization after surgery (days)	14 (6, 11–38)	20 (10, 15–28)	**0.028**
30-day mortality	1 (3.1%)	0	1.000

**Table 5:** Impact of reoperation on the hospitalization duration in the CR-PPAP and no-CR-PPAP groups.

Group	Reoperation	Duration of Hospitalization After the First Surgery (Days)	*p*
CR-PPAP (+)	Yes	15 (12–38, 10)	0.817
	No	14 (11–29, 5)	
CR-PPAP (–)	Yes	14 (11–22, 8)	0.132
	No	13 (8–25, 3)	

**Table 6:** CR-PPAP occurrence after PD in patients with and without neoadjuvant chemotherapy for pancreatic cancer.

	CR-PPAP (−) (*n* = 245, 86.9%)	CR-PPAP (+) (*n* = 37, 13.1%)	*p*
Neoadjuvant (−) (*n* = 146, 51.8%)	124 (84.9%)	22 (15.1%)	0.315
Neoadjuvant (+) (*n* = 136, 48.2%)	121 (89.0%)	15 (11.0%)	

**Table 7:** Risk factors of CR-PPAP after PD in patients with pancreatic cancer.

	*p*	OR	95%CI
BMI	0.993	1.001	0.890–1.125
MPD diameter	**<0.001**	0.328	0.195–0.551
Female sex	0.560	1.263	0.576–2.772
Soft pancreatic tissue	0.918	1.040	0.490–2.210
Surgery duration	0.169	1.005	0.998–1.012

## Data Availability

The data are available from the corresponding author on reasonable request.

## Abbreviations

The following abbreviations are used in this manuscript:

PPAP	Post-pancreatectomy Acute Pancreatitis
ISGPS	International Study Group of Pancreatic Surgery
CR-PPAP	Clinically Relevant Post-Pancreatectomy Acute Pancreatitis
BMI	Body Mass Index
ASA	American Society of Anesthesiologists
MPD	Main Pancreatic Duct
CRP	C-Reactive Protein
CT	Computed Tomography
PD	Pancreatoduodenectomy
POH	Postoperative Hyperamylasemia
CR-POPF	Clinically Relevant Postoperative Pancreatic Fistula
CD	Clavien–Dindo
OR	Odds Ratio
CI	Confidence Interval
LMWH	Low-Molecular-Weight Heparin
GM	Gabexate Mesylate
ERCP	Endoscopic Retrograde Cholangiopancreatography
DGE	Delayed Gastric Emptying
